# Malaria and dengue outbreaks during a national disaster in Pakistan: A rising concern for public health

**DOI:** 10.7189/jogh.12.03076

**Published:** 2022-12-03

**Authors:** Talal Arshad, Ali Wajahat, Adina Jabeen, Syed Hasan Ali

**Affiliations:** Dow Medical College, Dow University of Health Sciences, Karachi, Pakistan

Mosquitoes have been widely responsible for the spread of two major diseases in the underdeveloped world – malaria and dengue. Around 240 million instances of malaria were estimated globally as of 2020, of which 627 000 resulted in fatalities, according to the World Health Organization (WHO) [1]. On the other hand, WHO estimates that there are close to four hundred million dengue cases annually, three-fourths of which are from Asia [2]. These mosquito-borne diseases are endemic in Pakistan, with over 350 000 reported cases of malaria and 113 deaths reported in 2021. Notably, malaria is often misdiagnosed or underreported due to a lack of appropriate resources. As a result, WHO estimated over 956 000 cases of malaria occurred, resulting in 805 deaths [3]. Similarly, Pakistan reported approximately 49 000 dengue cases with 183 deaths in 2021 [4].

Though malaria and dengue fever have often remained under control in Pakistan, there have been major outbreaks in various situations, as per WHO reports. Dengue and malaria epidemics are common in the post-monsoon season due to prolonged periods of stagnant water bodies in most of the country, owing to poor sanitary and sewage systems. This year’s (2022) monsoon season has wreaked considerable havoc in Pakistan, as the recorded rainfall was three times the average annual rainfall (more than five times in the southern provinces of Sindh and Balochistan). The intense heat waves experienced this year, along with melting glaciers due to global warming, contributed to the destruction of dams leading to a huge rush of water and the resultant floods [5]. Millions of people have been affected by this calamity and over 1000 lives have been lost.

Consequently, a massive amount of stagnant water has accumulated around the country, functioning as a mosquito nursery. Since Pakistan is already in an economic crisis, and since the magnitude of this devastation is significant, the number of malaria and dengue cases is expected to exceed records. The outcome is expected to worsen, as Pakistan's health care system is already overburdened with COVID-19 patients.

## CHALLENGES

Pakistan is currently facing the worst floods of its history, as the spilling of rivers out of their banks, flash flooding, and glacial lakes bursting have submerged at least a third of the country [5]. This extreme hydro-meteorological disaster has displaced approximately 33 million persons, injured 12 867, claimed 1725 lives, and destroyed the basic infrastructure of a large part of the country, thereby creating a direct impact on the public health domain by endangering the lives of those affected by the flood [5,6]. Apart from the direct effects of the catastrophic flood itself, Pakistan faces several other challenges, especially in efficiently tackling the ruinous circumstances related to public health – particularly the rising cases of malaria and dengue infections:

1. This humanitarian crisis unveiled severe flaws in the authorities’ plans; inadequate flood warning systems, ineffective disaster management protocols, unchecked urban growth, and inadequate drainage and storage systems have majorly contributed to the emergence of vast water-logged land across Pakistan, serving as breeding grounds for various pathogens and vectors of water-borne diseases [5].
2. Many similar hydro-meteorological disasters in recent history have shown that the effects of floods on public health include outbreaks of mosquito-borne illnesses (like malaria and dengue) due to a rise in mosquito breeding grounds. These effects are accelerated by the influx of people into crowded settlements, especially in endemic countries like Pakistan [7,8].

**Figure:**
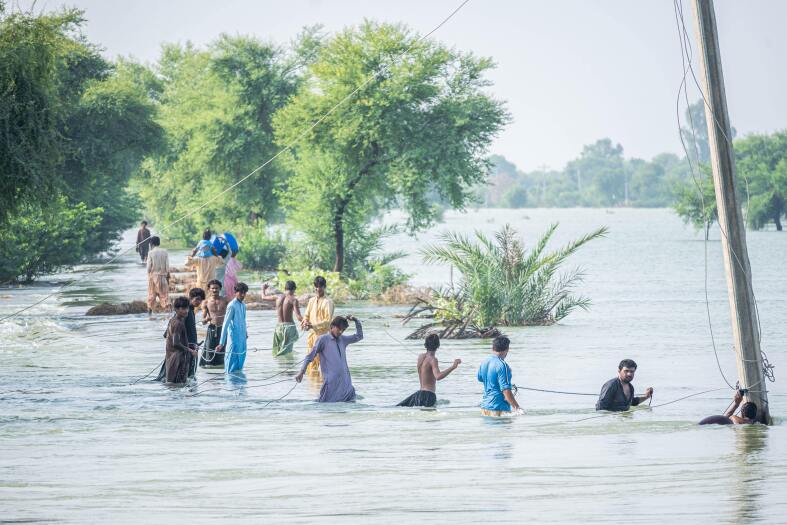
Photo: Flood-affected victims attempting to cross the deep water to rescue stranded members in Balochistan, Pakistan. Source: https://www.pexels.com/photo/people-walking-on-water-near-green-trees-13455964/). Free to use under Pexels license.
3. Pakistan's already struggling economy received another major blow in the form of this climatic catastrophe. Financial losses due to complete or partial damage to various infrastructures (including health facilities) across the country are officially estimated to exceed US$30 billion [9,10]. WHO reported that over 1460 healthcare facilities have been affected, 432 of which are completely damaged. Moreover, there is limited access to basic health facilities, healthcare professionals, and medical supplies, further deteriorating the already compromised public health in flood-affected regions [10]. Although Pakistan is receiving aid from various countries and organizations, considering the magnitude of this destruction, there is still a significant deficit in resource allocation.
4. According to early estimates, five million persons affected by the floods (including children) are highly susceptible to infections such as malaria and dengue, since immunization campaigns and other public health programs are in disarray. Consequently, millions of children are at an increased risk of morbidity and mortality from coinfections with already existing illnesses such as COVID-19, poliomyelitis, typhoid, and measles [10,11].
5. Unfortunately, no licensed malaria or dengue vaccines are officially in use. Instead, only preventive methods (mostly mosquito control) are implemented. These prove to be inadequate given the current widespread devastation, unavailability of efficient surveillance systems, and poorly developed diagnostic facilities, resulting in insufficient information about the magnitude of these outbreaks [12].
6. The catastrophe has not only had physical implications but can also have psychological effects; Chung et al. [13] found that victims of the 2010 flood reported signs of PTSD and psychiatric comorbidities. It is highly probable that the affected persons may now also display these symptoms.

Evaluating this alarming situation and consequent health risks, WHO has ascertained that the affected populations face serious threats to their health, primarily due to the possible extensive spread of malaria and dengue [14]. Amidst the enormous physical destruction caused by this unprecedented flooding, the outbreaks of malaria and dengue have posed a major risk of a potential secondary disaster in the form of a countrywide public health emergency.

## EFFORTS AND RECOMMENDATIONS

Before the 2022 monsoon season, Pakistan was employing strategies suggested by the WHO to keep mosquito-borne diseases under control. The Directorate of Malaria Control (DoMC) provided free long-lasting insecticidal nets (LLINs) to prevent mosquito bites. The government has also been providing free-of-cost diagnostic tests and medications such as chloroquine and primaquine. Furthermore, indoor residual spraying and larval control are also being adopted. These measures are chiefly funded by global funds and the government itself [3]. In cases of outbreaks, several response departments have also been formed to survey the situation in areas where outbreaks are frequent [15]. Currently, the National Disaster Management Authority (NDMA) and the Flood Forecasting division (a branch of the Pakistan Meteorological Department) have initiated efforts to prevent further destruction; for example, Manchar lake has reached dangerously high levels, so the government has started breaching the lake and has called for the evacuation of nearby residents [16].

Despite all the government’s efforts to provide relief to flood victims, there seems to be a gap in preparation for a potential surge of water-related illnesses that could manifest as a secondary disaster. The outbreaks could claim further lives and put more pressure on the already overburdened health care system. To avoid such aggravations, the following set of recommendations may be followed:

1. Pakistan possesses a subpar infrastructure, largely due to cities getting overcrowded without the current framework being developed further, exacerbating the strain on the existing inadequate drainage facilities [17]. Therefore, it is necessary that the government allocates more budget towards preparing the country’s infrastructure to withstand such disasters by building flood dams, channel modifications, and floodwalls. This is especially important since more hydro-meteorological disasters are expected in the future due to global warming and the consequent melting of glaciers [17].
2. Pakistan’s Health Ministry’s budget received a massive downsize this year (2022) as it was reduced from 158 billion Pakistani Rupees to 19 billion. This may consequently deplete the services provided by the healthcare system, especially when dealing with national public health emergencies [18]. Hence, the government should increase the healthcare budget to handle such situations effectively. Also, Pakistan needs support from international humanitarian organizations such as WHO and UNICEF in terms of finance, trained staff, and resources to tackle such outbreaks. This would help the government with supplying the public with more insecticide-treated bed nets (ITNs)/LLINs, indoor spraying, medications, and diagnostic tests.
3. Pakistan needs to improve its surveillance of water-borne diseases and conduct long-term research on the efficacy of treatments and preventative measures currently being employed. As stated in the Malaria 2021 Country Profile for Pakistan, adequate active case detection (ACD) and mass screening policies have yet to be undertaken [3]. The health ministry should form a competent rapid response division, well-equipped to act swiftly to contain the disease outbreak.
4. Pakistan suffers from a paucity of several pharmaceutical prophylactic solutions for malaria and dengue. For example, Pakistan does not have commercially available dengue vaccines [15]. The provision of vaccines and preventative treatments will help substantially reduce the prevalence of these diseases.
5. Spreading awareness and guidance through caller tune and SMS services, as well as electronic media advertisements, could also help less affected areas where people have access to the internet. This same strategy has been adopted by several telephone networks such as Zong 4G, in collaboration with UNICEF Pakistan, to create awareness about COVID-19 and its vaccines [19].
6. The government should work towards rehabilitating displaced individuals and setting-up health centres equipped with medical personnel (especially a psychiatrist or a psychologist) to alleviate their mental and emotional burdens. Similar strategies have been employed and have proved beneficial, previously in Freetown, Sierra Leone, to provide psychological first aid (PFA) to those affected by the flooding and mudslides that occurred in 2017 [20].

## CONCLUSION

The current efforts to control the spread of vector-borne diseases such as malaria and dengue have been insufficient. Pakistan’s population remains vastly vulnerable to contracting malaria and dengue [21]. The country’s current circumstances (ie, floods and the economic crisis) have further impaired the government’s ability to appropriately and timely respond to the needs of those affected. It is imperative to allocate global support through finance, trained staff, and resources to aid the victims and improve control and surveillance measures, without which further losses of lives are inescapable. Prompt steps are to be taken on national and international levels to alleviate the burden of malaria and dengue in Pakistan.
